# Age, gender, and financial literacy in Japan

**DOI:** 10.1371/journal.pone.0259393

**Published:** 2021-11-17

**Authors:** Shohei Okamoto, Kohei Komamura

**Affiliations:** 1 Research Team for Social Participation and Community Health, Tokyo Metropolitan Institute of Gerontology, Itabashi-ku, Tokyo, Japan; 2 Institute for Global Health Policy Research, National Center for Global Health and Medicine, Shinjuku-ku, Tokyo, Japan; 3 Research Center for Financial Gerontology, Institute for Economics Studies, Keio University, Minato-ku, Tokyo, Japan; 4 Faculty of Economics, Keio University, Minato-ku, Tokyo, Japan; Politecnico di Torino, ITALY

## Abstract

**Objective:**

The aim of this study is to investigate the association between financial literacy and age as well as gender differences in financial literacy.

**Methods:**

We analyse a sample of 25,000 individuals from ‘The Financial Literacy Survey 2016’ conducted by the Central Council for Financial Services Information (Bank of Japan). The analysis focuses on the relationship of age and financial literacy as well as that of age and self-rated financial knowledge. To consider factors accounting for gender differences in financial literacy, we use the Blinder-Oaxaca decomposition method. To further our understanding of financial literacy, we conduct additional analyses on financial behaviour and attitude.

**Results:**

Although age is associated with increased financial literacy (Men, β: 0.249, standard error [SE]: 0.030; Women, 0.354, SE: 0.026), the growth rate decreases among the older respondents (Men, β: -0.002, SE: 0.000; Women, -0.003, SE: 0.000). However, the association between age and self-rated financial knowledge among men moves in the opposite direction (Age, β: -0.021, SE: 0.009, Age^2^, β: 0.000, SE: 0.000). Furthermore, female respondents are likely to be less financially literate than their male counterparts (β: -0.586, SE: 0.095) due to gender differences in the distribution of the factors that affect financial literacy (specifically education), their responses to financial literacy, and the interactions of these effects. In contrast to knowledge-based financial literacy, financial behaviour and attitudes among women are more preferable to those among men, namely, more premeditated.

**Conclusion:**

Financial literacy increases until about one’s early 60s, after which it declines, while confidence in financial literacy reflects the inverse trend, especially among men. Additionally, men are more financially literate than women; however, these differences could be mitigated through education. Meanwhile, financial behaviour and attitudes among men are less premeditated. Thus, policies are needed that can help older adults with their financial decision-making, enhance women’s financial literacy, and improve men’s financial behaviours and attitudes.

## Introduction

### Financial literacy in Japan

In many industrialised countries faced with ageing populations, policies that can optimise costs associated with ageing (i.e. public pensions) have become increasingly important. In this environment, an individual’s ability to accumulate savings and build assets are fundamental to living a longer life, occasionally described as ‘the 100-Year Life’.

In Japan, new schemes, such as the Nippon Individual Savings Account (NISA) and the individual-type defined contribution pension plan (iDeCo), have been created to encourage individuals to build assets independently. The Financial Service Agency in Japan report that the number of NISA accounts and the purchase price increased from 7.27 million accounts and 1 trillion 56 billion JPY, respectively, as of June 2014 to 10.37 million accounts and 8 trillion 37 billion JPY, respectively, as of June 2016 [1]. To take advantage of these schemes, individuals need financial knowledge and the ability to make financially sound behavioural choices. However, in Japan, financial literacy is lower than in other industrialised countries [2]. Since the Japanese have one of the longest average life spans in the world, the financial literacy of the nation needs to improve to promote proper financial behavioural choices; and research in this area should be encouraged.

### Determinants of financial literacy

Most literature on this topic focuses on the determinants of financial literacy as well as the association between financial literacy and outcomes related to financial decision making (for a review: e.g., Lusardi and Mitchell [3]). In particular, there have been many studies on the relationship between financial literacy and financial behavioural choices. As the studies have shown, financial literacy is associated with a higher probability of investment in financial assets [4,5]. Furthermore, such knowledge results in an increased ability to accumulate assets [6,7], but is also affected by the possession of financial assets and experience related to finance [8]. Some studies also suggest that those with financial literacy are likely to have retirement plans [4,5,9,10]. Thus, financial literacy has been shown to be useful for asset accumulation by influencing proper financial behavioural choices and consumption smoothing at each life stage.

Globally, there is accumulating evidence on the heterogeneity in financial literacy by age, gender, education, income, and employment status [3]. Based on a theoretical framework for financial literacy, optimal paths for financial knowledge are hump shaped over one’s life cycle, which is supported by empirical findings that financial literacy is the lowest among the young and those who are older. Disparities in financial literacy by gender (i.e. men are more financially literate than women) seem ubiquitously present across the world. At least before widowhood, a division of labour where wives invest less in education, occupation, and financial knowledge may partly explain women’s lower financial literacy [11].

Previous research has examined Japanese samples and investigated the association of financial literacy, individual characteristics, and financial behavioural choices [12–16]. These scholars report similar results to studies in other countries: namely, men and those with higher education and income tend to have higher financial literacy; moreover, financial literacy is positively correlated with participation in investment activities and retirement plans. In Japan, the conceptualisation of gender roles has restricted women’s participation in education and occupation for decades [17]. Although the situation has improved recently, disparities in higher education and occupation across gender still exist [18]. Therefore, gender inequality in socioeconomic status may be a significant contributing factor to male-female disparities in financial literacy, considering that socioeconomic factors, such as education and occupation, are important determinants of financial literacy [3].

To identify policy implications that can enhance financial literacy, it is essential to gain a better understanding of those who need support in their financial decision making and how this can be achieved. Therefore, this paper aims to fill this gap in the research and contribute to the understanding of financial literacy in Japan by investigating the association between financial literacy and individual characteristics (i.e. age and gender). To that end, we test three hypotheses:

*Hypothesis 1*: *Financial literacy increases with age but reaches a peak at some age point*.
*Hypothesis 2*: *There are gender gaps in financial literacy*.
*Hypothesis 3*: *Gender gaps in financial literacy can be explained partly by factors that are distributed differently among men and women (e*.*g*. *education level and employment)*.

We extend the existing research findings in the field in two ways. First, we analyse differences associated with age in terms of financial literacy. As the sample in this study contains individuals between 18 and 79 years old, we are able to draw associations between how financial literacy differs with age according to the sample. In Japan today, most financial assets are owned by seniors. Therefore, it is meaningful to investigate the association of financial literacy with age. In many previous studies in Japan, only samples of university students or seniors have been used. Moreover, financial literacy has been measured by few questions, which only capture a small variation, and determinants of financial literacy have not been well modelled through regression analysis.

Second, we analyse financial literacy in terms of gender differences. As Lusardi and Mitchell [3] indicate, the reasons for gender gaps are not explicitly known. Thus, this study examines gender differences in financial literacy by using a decomposition method.

While studies in Japan [15,16] investigate demographic and socioeconomic determinants of financial literacy using the same dataset that we use in the current study, we further assess the discrepancies between objectively measured financial literacy and the self-assessed financial knowledge as well as factors that contribute to gender differences in financial literacy.

## Methods

### Data

Our study uses a sample from The Financial Literacy Survey 2016 conducted by the Central Council for Financial Services Information (Bank of Japan). The Financial Literacy Survey 2016 was an internet survey conducted in February and March 2016 to investigate the level of financial literacy among Japanese people. The sample comprises 25,000 individuals between 18 and 79 years old from all prefectures in proportion to the Japanese demographic structure. Ethical approval was not required since this study was based on secondary analysis of publicly available data.

### Definitions of variables

#### Dependent variables

Financial literacy denotes not merely financial understanding (i.e. financial knowledge and education), but also practical management of financial assets, which is considered helpful for individuals in accumulating assets [19,20]. A previous study suggests that there are three important components of financial literacy that focus on understanding [3]: (1) numeracy, or the capacity to calculate a simple compound interest rate; (2) understanding inflation; and (3) knowledge about stocks, stock mutual funds, and risk diversification.

For our analysis, we measured knowledge-based financial literacy by the number of correct answers out of 25 questions on the survey (Table 1). The items measure multiple dimensions of financial literacy as they contain a wide range of topics (e.g. financial knowledge and norms), including items used in previous studies [3,21,22].

**Table 1:** A list of questions to measure knowledge-based financial literacy.

1	Which of the following statements on household behaviour is inappropriate?
2	Which of the following statements on family budget management and credit cards is inappropriate?
3	Taro and Hanako are the same age. At age 25 Hanako began saving 100,000 yen per year and continued to save the same amount annually thereafter. Meanwhile, Taro did not save money at age 25, but began saving 200,000 yen per year at age 50. When they are aged 75, which of them will have more money saved?
4	What are the so-called three major expenses in life?
5	Which of the following is inappropriate as an action to take when concluding a contract?
6	Which of the following is inappropriate as a behaviour to avoid being involved in financial trouble?
7	Which of the following is inappropriate as an action related to Internet transactions?
8	Suppose you put 1 million yen into a savings account with a guaranteed interest rate of 2% per year. If no further deposits or withdrawals are made, how much would be in the account after 1 year, once the interest payment is made?
9	Then, how much would be in the account after 5 years?
10	Imagine that the interest rate on your savings account was 1% per year and inflation was 2% per year. After 1 year, how much would you be able to buy with the money in this account?
11	High inflation means that the cost of living is increasing rapidly [T or F]
12	When compared, a 15-year mortgage typically requires higher monthly payments than a 30-year loan, but the total interest paid over the life of the loan will be less [T or F]
13	An investment with a high return is likely to be high risk [T or F]
14	Buying a single company’s stock usually provides a safer return than a stock mutual fund [T or F]
15	If interest rates rise, what will typically happen to bond prices?
16	Which of the following is appropriate as an action to take when investing (making deposits, etc.) or borrowing funds at a time of interest rate rise?
17	Which of the following statements on the basic function of insurance is appropriate?
18	When a 50-year-old man reviews his life insurance policy (whole life insurance) after his children have become financially independent, which of the following statements is appropriate?
19	Which of the following statements on insurance is inappropriate?
20	Which of the following statements on mortgages is appropriate?
21	Suppose you owe 100,000 yen on a loan and the interest rate you are charged is 20% per year compounded annually. If you didn’t pay anything off, at this interest rate, how many years would it take for the amount you owe to double?
22	Which of the following statements on the types of deposits protected up to 10 million yen under Japan’s deposit insurance system is appropriate?
23	Which of the following is inappropriate as behaviour or attitude when determining whether to purchase an unfamiliar financial product?
24	Which of the following is appropriate as an action to take when considering purchase of a financial product with a complicated structure?
25	Which of the following is inappropriate as a consultant office or a system to be used when trouble occurs in relation to a contract for a financial product?

Table 1 shows a list of questions used in the Financial Literacy Survey 2016 to measure financial literacy. The information was cited from the web page of The Central Council for Financial Services Information: https://www.shiruporuto.jp/e/survey/ (accessed: 30 April, 2020).

In addition to measuring financial literacy, we assess the association between age and self-rated financial literacy to evaluate how our definition of financial literacy and self-rated financial literacy diverge with age, considering that previous literature indicates that people tend to overestimate their level of financial literacy [3]. Self-rated financial literacy is determined by a five-point Likert scale from ‘very high’ to ‘very low’ for the question, ‘What level is your general knowledge about finance compared with others?’

To complement the understanding of financial literacy measured mainly by financial knowledge, we focus additionally on financial behaviours and attitudes, which can contribute to positive outcomes of financial literacy [15,23]. Financial behaviour measures people’s action in a financial transaction, which can be derived from financial knowledge. In contrast, financial attitude, as asked in the survey, measures the individual’s time preference for the transaction. Those who put too much value on the present and less on the future may suffer from a shortage of financial assets in the long run due to a lack of appropriate financial planning and management. Thus, an excessive discount of future benefits can be regarded as a negative in financial decision making, at least for younger people.

Following the definitions in a previous study [15], we use the following qualitative questions to measure financial behaviours and attitudes.

Financial behaviour:

1. Before I buy something I carefully consider whether I can afford it.
2. I pay my bills on time.
3. I set long term financial goals and strive to achieve them.
4. I keep a close personal watch on my financial affairs.

Financial attitude:

1. I find it more satisfying to spend money than to save it for the long term.
2. I tend to live for today and let tomorrow take care of itself.
3. If I had the choice of (1) receiving 100,000 yen now or (2) receiving 110,000 yen in 1 year, I would choose (1), provided that I can definitely receive the money.

Respondents are asked to rate each item on a five-point Likert scale, where 1 = agree and 5 = disagree. Aggregating the responses, we create scores to measure financial behaviours and attitudes; these are transformed so that higher values represent more preferable financial behaviours and attitudes, namely, premeditated financial considerations.

#### Independent variables

The explanatory variables comprise individual demographic and socioeconomic factors that affect financial literacy: the age of the respondent, the square of the age, educational attainment, occupation, financial education, household income, and financial assets: Education is categorised into three groups: high-school graduate or lower; vocational college, junior college or equivalent; and university graduate or higher. Occupation is divided into five categories: employee or public official, self-employed, part-time, not employed (including domestic worker), and others. Financial education is a binary dummy variable, which takes a value of one if a respondent has received any financial education (e.g. about life planning and management of a family budget) and zero otherwise. Household income denotes the gross annual household income from the previous year, including revenues from all assets. Household income was not equalised as the information on the number of household members was not available. The financial assets refer to the amount of financial assets (e.g. savings and stock) that a household has at the time of the survey.

### Empirical strategy

#### Financial literacy and individual characteristics

We begin by investigating financial literacy, self-rated financial literacy, and age to test hypothesis 1. We use the ordinary least squares method for the analysis of financial literacy and the ordered logistic regression for self-rated financial literacy.

#### Gender differences in financial literacy

To test hypotheses 2 and 3 (gender gaps in financial literacy), we use the Blinder-Oaxaca decomposition [24,25]. The decomposition model is formulated as follows:

$$Y_{i}=\underset{j}{\sum }\;\beta_{j}X_{ji}+\varepsilon_{i}\;i=1,\ldots ,n;j=2,\ldots ,k$$

where *Y*_*i*_ denotes the financial literacy of individual *i*, *X*_*ji*_ represents independent variables (e.g. age and education) for individual *i*, and *ε*_*i*_ is a stochastic disturbance. *β*_*j*_ is the parameter to be estimated.

Assuming E (*β*_*j*_) = *β*_*j*_ and E (*ε*_*j*_) = 0, the estimations of the subsample means for women and men, respectively, are:

$${\bar{Y}}_{\text{j}}^{\text{F}}=\sum_{j}b_{j}^{F}{\bar{X}}_{\text{j}}^{\text{F}}$$

$${\bar{Y}}_{\text{j}}^{\text{M}}=\sum_{j}b_{j}^{M}{\bar{X}}_{\text{j}}^{\text{M}}$$

F and M indicate women and men, respectively, and $\overset{-}{Y}$ and $\overset{-}{X}$ denote the sample means.

Thus, the difference in the sample means of men and women is given by:

$$\begin{array}{ll} {\bar{Y}}_{\text{j}}^{\text{M}}-{\bar{Y}}_{\text{j}}^{\text{F}} & =\sum_{j}b_{j}^{M}{\bar{X}}_{\text{j}}^{\text{M}}-\sum_{j}b_{j}^{F}{\bar{X}}_{\text{j}}^{\text{F}}\\ & =\sum_{j}b_{j}^{F}({\bar{X}}_{\text{j}}^{\text{M}}-{\bar{X}}_{\text{j}}^{\text{F}})+\sum_{j}(b_{j}^{M}-b_{j}^{F}){\bar{X}}_{\text{j}}^{\text{F}}+\sum_{j}(b_{j}^{M}-b_{j}^{F})({\bar{X}}_{\text{j}}^{\text{M}}-{\bar{X}}_{\text{j}}^{\text{F}}) \end{array}$$ (1)

Here, differences in financial literacy between men and women can be decomposed into three components: the endowment, coefficient, and interaction effects. The endowment effect denotes the difference in the distribution between men and women of the explanatory variables that affect financial literacy, while the coefficient effect refers to the gender difference in the responses of the explanatory variables to the outcome. The interaction effect captures the existence of both the endowment and coefficient effects, including differences in the intercept.

To test hypothesis 2, we use the significance of the coefficient of the female dummy variable in the model testing hypothesis 1. Next, we decompose the gender differences in financial literacy to test hypothesis 3 using the Blinder-Oaxaca decomposition model as shown in Eq (1).

For financial behaviours and attitudes, we adopt the same procedures as the estimates for financial literacy. To account for the fact that financial knowledge can affect financial behaviour and attitude, we include financial literacy as a covariate in the models as well.

We exclude students (n = 1,212) from our analyses, since they may not be financially independent. Among the remaining 23,788 respondents, there are some whose educational attainment is unknown (n = 37), some who did not score their own financial literacy (n = 614), and some who lacked income information (n = 4,204) or financial asset information (n = 3,705). Thus, the final usable number of complete cases equalled 15,228 individuals. Table 2 shows the descriptive statistics by gender. The proportions of employees/public officials and self-employed individuals are higher among men, while the proportions of part-time workers and those not working are higher among women. Furthermore, more men than women graduated from university or higher and received financial education.

**Table 2:** Descriptive statistics for complete cases.

		N	Mean/proportion	Standard deviation
Financial literacy		15,228	15.2	6.6
Financial behaviour		15,228	15.6	2.8
Financial attitude		15,228	9.7	2.8
Experience of investment		15,228	0.158	0.365
Age		15,228	50.4	15.5
Male		15,228	0.529	0.499
Occupation	Employee/public official	15,228	0.414	0.493
	Self-employed	15,228	0.073	0.260
	Part-time	15,228	0.132	0.339
	Not working	15,228	0.362	0.481
	Others	15,228	0.018	0.133
Education	High school or lower	15,228	0.342	0.474
	Junior college or equivalent	15,228	0.209	0.407
	University or higher	15,228	0.449	0.497
Financial education		15,228	0.074	0.262
Household income (10,000 JPY)		15,228	515.293	336.294
Financial assets (10,000 JPY)		15,228	744.065	744.266
		Men (n = 8,054)	Women (n = 7,174)	P-value
Age		50.2	50.6	p = 0.22
Occupation	Employee/public official	0.580	0.229	p<0.01
	Self-employed	0.102	0.040	
	Part-time	0.066	0.207	
	Not working	0.234	0.506	
	Others	0.019	0.018	
Education	High school or lower	0.296	0.395	p<0.01
	Junior college	0.124	0.304	
	University or higher	0.580	0.301	
Financial education		0.091	0.055	p<0.01
Household income (10,000 JPY)		540.4	487.1	p<0.01
Financial assets (10,000 JPY)		758.3	728.1	P<0.05

a) Numbers are sample means or proportions.

b) Welch t-test for continuous variables and χ^2^ test for categorical variables.

To address the issue of potential non-response bias, we adopt multiple imputation for missing variables by the missing at random assumption and predictive mean matching using variables of financial attitudes, behaviours, and knowledge, adding 20 imputations. All analyses are performed using Stata version 17.0.

## Results

### Financial literacy

Table 3 shows the results for financial literacy. Men tended to be more financially literate than women (β: -0.586, standard error [SE]: 0.095); however, the tendencies between men and women were similar: age was associated with an increase in financial literacy (Men, β: 0.249, SE: 0.030; Women, 0.354, SE: 0.026) but the growth rate turned downward among the oldest respondents (Men, β: -0.002, SE: 0.000; Women, -0.003, SE: 0.000).

**Table 3:** Estimation results for financial literacy by OLS.

	(1) All	(2) Men	(3) Women
Age	0.297**	0.249**	0.354**
	(0.020)	(0.030)	(0.026)
Age^2^	-0.002**	-0.002**	-0.003**
	(0.000)	(0.000)	(0.000)
Female	-0.586**		
	(0.095)		
Occupation: employee/public official	Reference		
Self-employed	-0.322	-0.275	-0.498
	(0.177)	(0.215)	(0.318)
Part-time	-0.331*	-0.715**	-0.371*
	(0.139)	(0.252)	(0.178)
Not working	0.056	0.350	-0.216
	(0.122)	(0.208)	(0.162)
Others	0.663*	0.475	0.716
	(0.286)	(0.409)	(0.402)
Education: high school or lower	Reference		
Junior college	0.287*	0.067	0.411**
	(0.113)	(0.208)	(0.135)
University or higher	2.222**	1.935**	2.490**
	(0.103)	(0.146)	(0.146)
Financial education	0.820**	0.211	1.751**
	(0.194)	(0.271)	(0.276)
Household income/100	0.139**	0.157**	0.127**
	(0.016)	(0.024)	(0.021)
Financial assets/100	0.207**	0.203**	0.211**
	(0.008)	(0.010)	(0.011)
Constant	2.611**	3.798**	0.805
	(0.457)	(0.718)	(0.596)
N	23,788	11,658	12,130
Number of imputations	20		

a) coefficients, and robust standard errors in parentheses.

b) ** p<0.01, * p<0.05.

Table 4 presents the results of the self-rated financial literacy. While we found similar results to those for financial literacy, among men, the association with age at the tail end moved in the opposite direction (Age, β: -0.021, SE: 0.009, Age^2^, β: 0.000, SE: 0.000), namely, self-rated financial knowledge increased the most among the older male respondents.

**Table 4:** Estimation results for self-rated financial knowledge by ordered logistic regression.

	(2) Men	(3) Women
Age	-0.021*	0.055**
	(0.009)	(0.009)
Age^2^	0.000**	-0.000**
	(0.000)	(0.000)
Occupation: employee/public official		
Self-employed	0.004	-0.056
	(0.061)	(0.095)
Part-time	-0.348**	-0.287**
	(0.075)	(0.056)
Not working	-0.325**	-0.270**
	(0.062)	(0.051)
Others	-0.338*	-0.102
	(0.134)	(0.140)
Education: high school or lower		
Junior college	-0.021	0.052
	(0.059)	(0.043)
University or higher	0.347**	0.154**
	(0.041)	(0.046)
Financial education	0.904**	0.988**
	(0.073)	(0.089)
Household income/100	0.037**	0.017**
	(0.007)	(0.006)
Financial assets/100	0.071**	0.075**
	(0.003)	(0.003)
/cut1	-1.601**	0.233
	(0.218)	(0.196)
/cut2	0.081	1.999**
	(0.217)	(0.198)
/cut3	2.557**	4.742**
	(0.218)	(0.203)
/cut4	5.046**	7.680**
	(0.229)	(0.236)
N	11,658	12,130
Number of imputations	20	

a) coefficients, and robust standard errors in parentheses.

b) ** p<0.01, * p<0.05.

**Fig 1:**
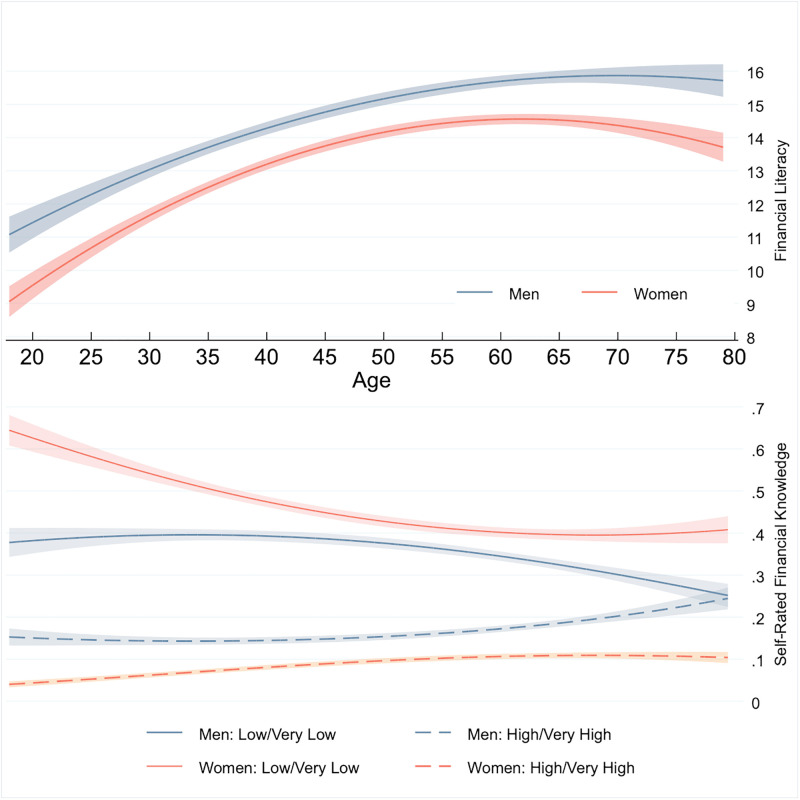
Age, financial literacy, and self-rated financial knowledge. This figure presents the relationship of age with financial literacy and self-rated financial knowledge based on the estimation results in Table 2 for financial literacy. For self-rated financial literacy, we obtain marginal effects of age and the square of age from ordered logistic regression with the outcomes of self-rated financial knowledge in a three-point Likert scale (very low/low, fair, and high/very high) for presentation simplicity of the results. The lines show point estimates, with shaded areas representing 95% confidence intervals, estimated by robust standard errors.

Fig 1 represents the association of age and financial literacy and age and self-rated financial knowledge using the previous estimations. The financial literacy trend shows an increase with age, which peaks when both men and women are in their early 60s. However, self-rated financial literacy, particularly for men, appears the lowest around age 70 and then begins to increase among those older than that, in contrast to the trend for the measured financial literacy.

### Gender gaps in financial literacy

The results indicate that men are more financially literate than women, with the coefficient of the female dummy variable significant and negative (Table 1). Thus, the hypothesis that there are gender gaps in financial literacy is supported.

Table 5 lists the result for the decomposition analysis of the gender gaps in financial literacy. The observed gender gaps are explained by the differences in the distribution of the explanatory variables (64.1%), the responses for the explanatory variables (53.7%), and their interactions (-17.8%). Among endowment effects, which account for the largest contribution among the three different effects, women’s lower financial literacy is explained mainly by the smaller number of women who graduated with a university degree or higher (85.1%), followed by age, occupation as a part-time worker, receiving financial education, and income.

**Table 5:** Decomposition of gender differences in financial literacy.

Financial Literacy	Men: 14.719 (0.067) Women: 13.480 (0.060)	Difference		
		1.240** (0.090)		
		Endowments	Coefficients	Interaction
Overall		0.794**(0.073)	0.666**(0.113)	-0.220*(0.099)
Attribution		64.1%	53.7%	-17.8%
Age		-0.192**(0.073)	-5.302**(2.014)	0.057(0.030)
		-24.2%	-796.5%	-25.8%
Age^2^		0.157**(0.061)	3.017**(1.130)	-0.059(0.031)
		19.8%	453.3%	26.9%
Occupation: employee/public official				
Self-employed		-0.034(0.021)	0.009(0.015)	0.015(0.025)
		-4.2%	1.3%	-6.6%
Part-time		0.050*(0.024)	-0.073(0.064)	0.046(0.041)
		6.2%	-11.0%	-20.9%
Not working		0.058(0.043)	0.288*(0.136)	-0.151*(0.071)
		7.2%	43.3%	68.3%
Others		0.001(0.002)	-0.005(0.011)	-0.000(0.001)
		0.2%	-0.7%	0.2%
Education: high school or lower				
Junior college		-0.073**(0.024)	-0.106(0.076)	0.061(0.044)
		-9.1%	-15.9%	-27.5%
University or higher		0.676**(0.043)	-0.151**(0.058)	-0.144**(0.056)
		85.1%	-22.6%	65.5%
Financial education		0.061**(0.013)	-0.084**(0.022)	-0.054**(0.016)
		7.7%	-12.6%	24.6%
Household income/100		0.055**(0.011)	0.139(0.163)	0.012(0.014)
		6.9%	20.9%	-5.5%
Financial assets/100		0.035(0.023)	-0.073(0.108)	-0.002(0.003)
		4.4%	-10.9%	0.9%
Constant			3.005**(0.930)	
			451.5%	
N		23,788 (Men: 11,658, Women: 12,130)		
Number of imputations		20		

a) coefficients, and robust standard errors in parentheses.

b) ** p<0.01, * p<0.05.

### Financial behaviours and attitudes

Table 6 shows the results for financial behaviour. Women tend to reflect more preferable financially behaviour than men (β: 0.329, standard error [SE]: 0.040); however, the tendencies between men and women are similar: age is associated with a decrease in preferable financial behaviour (Men, β: -0.045, SE: 0.013; Women, -0.025, SE: 0.012) but the growth rate increases among the older respondents (Men, β: 0.001, SE: 0.000; Women, 0.000, SE: 0.000).

**Table 6:** Estimation results for financial behaviour by OLS.

	(1) All	(2) Men	(3) Women
Age	-0.032**	-0.045**	-0.025*
	(0.009)	(0.013)	(0.012)
Age^2^	0.000**	0.001**	0.000*
	(0.000)	(0.000)	(0.000)
Female	0.329**		
	(0.040)		
Occupation: employee/public official	Reference		
Self-employed	0.050	0.001	0.133
	(0.071)	(0.085)	(0.132)
Part-time	-0.042	-0.150	0.045
	(0.060)	(0.103)	(0.080)
Not working	0.140**	-0.015	0.251**
	(0.052)	(0.084)	(0.071)
Others	0.234	0.237	0.240
	(0.124)	(0.172)	(0.178)
Education: high school or lower	Reference		
Junior college	0.038	0.087	0.020
	(0.048)	(0.083)	(0.061)
University or higher	0.103*	0.070	0.134*
	(0.043)	(0.059)	(0.064)
Financial education	0.252**	0.232*	0.274*
	(0.075)	(0.094)	(0.118)
Financial literacy	0.076**	0.084**	0.067**
	(0.003)	(0.004)	(0.004)
Household income/100	-0.027**	-0.026*	-0.033**
	(0.007)	(0.010)	(0.009)
Financial assets/100	0.057**	0.063**	0.053**
	(0.003)	(0.005)	(0.005)
Constant	14.421**	14.548**	14.731**
	(0.203)	(0.308)	(0.276)
N	23,788	11,658	12,130
Number of imputations	20		

a) coefficients, and robust standard errors in parentheses.

b) ** p<0.01, * p<0.05.

Table 7 presents the results for financial attitude. Women are less likely than men to place a larger value on present benefits (β: 0.639, standard error [SE]: 0.039). The association with age is found only in women (Age, β: 0.035, SE: 0.012; Age^2^, -0.001, SE: 0.000).

**Table 7:** Estimation results for financial attitude by OLS.

	(1) All	(2) Men	(3) Women
Age	-0.027**	-0.013	0.035**
	(0.008)	(0.012)	(0.012)
Age^2^	0.000**	0.000	-0.001**
	(0.000)	(0.000)	(0.000)
Female	0.639**		
	(0.039)		
Occupation: employee/public official	Reference		
Self-employed	0.278**	0.308**	-0.320*
	(0.069)	(0.081)	(0.135)
Part-time	0.270**	0.418**	-0.239**
	(0.057)	(0.097)	(0.077)
Not working	-0.066	0.112	0.122
	(0.050)	(0.080)	(0.068)
Others	-0.158	-0.249	-0.007
	(0.128)	(0.177)	(0.187)
Education: high school or lower	Reference		
Junior college	-0.162**	-0.215**	0.116*
	(0.047)	(0.079)	(0.059)
University or higher	-0.175**	-0.184**	0.117
	(0.042)	(0.057)	(0.064)
Financial education	0.147*	0.125	-0.207
	(0.073)	(0.092)	(0.113)
Financial literacy			
Household income/100	-0.066**	-0.057**	0.076**
	(0.003)	(0.004)	(0.004)
Financial assets/100	0.024**	0.029**	-0.025**
	(0.006)	(0.009)	(0.009)
Constant	7.678**	7.952**	8.233**
	(0.190)	(0.280)	(0.267)
N	23,788	11,658	12,130
Number of imputations	20		

a) coefficients, and robust standard errors in parentheses.

b) ** p<0.01, * p<0.05.

**Fig 2:**
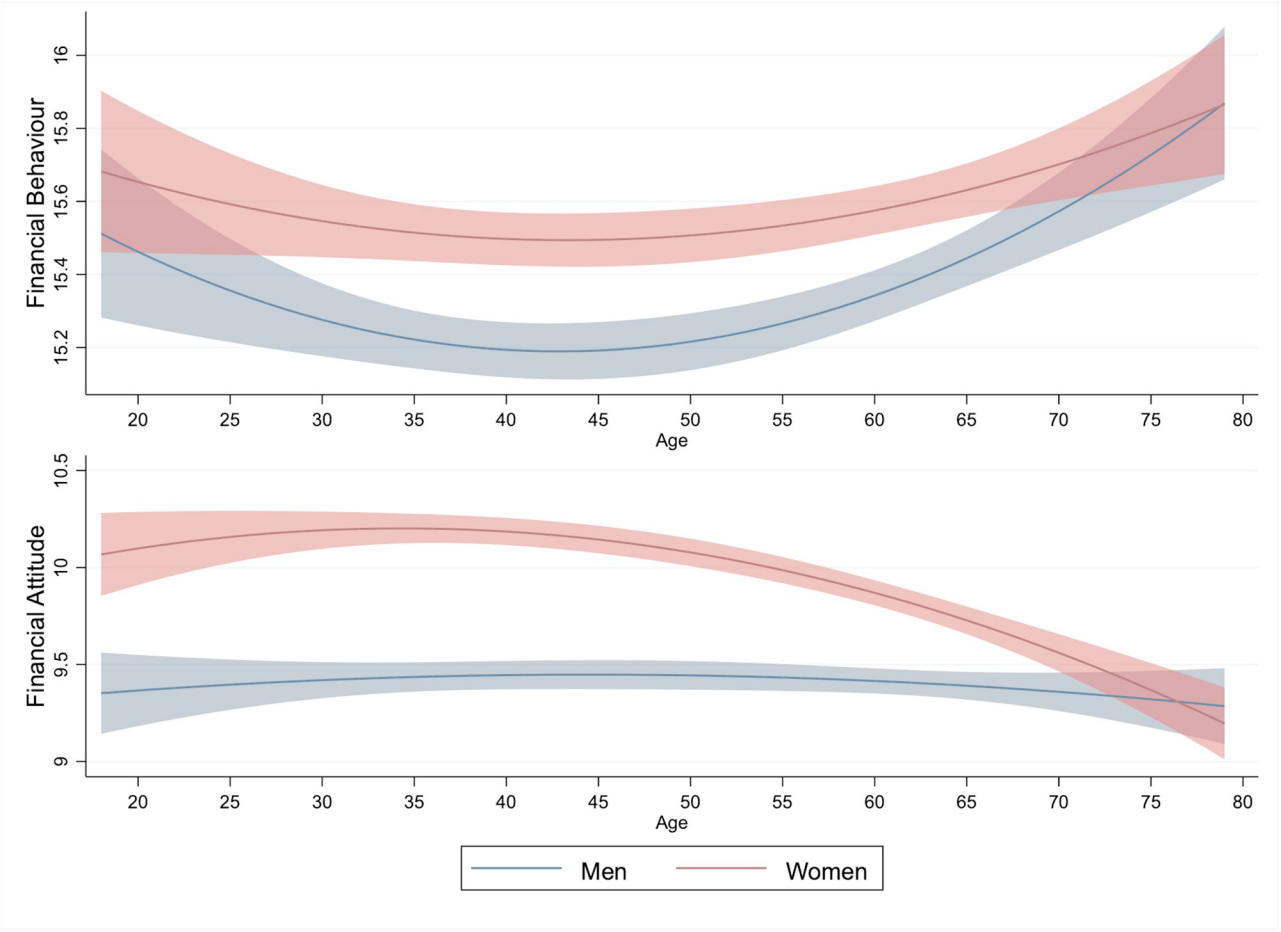
Age, financial behaviour, and financial attitude. This figure presents the relationship of age with financial behaviour and attitude based on the estimation results in Tables 6 and 7. The lines show point estimates, with shaded areas representing 95% confidence intervals estimated by robust standard errors.

Fig 2 represents the association of age with financial behaviour and attitude using the previous estimations. In contrast to financial literacy, the preferable financial behaviour trend shows a U-shaped curve with the lowest value around age 45 and higher values among women. For financial attitude, it is associated with age only in women, and shows a decrease with age after around age 35.

### Gender gaps in financial behaviours and attitudes

As shown in the results in Tables 6 and 7, we find that financial behaviour and attitude vary between men and women: In contrast to financial literacy measured by financial knowledge, women tend to reflect more preferable financial behaviours and attitudes than men. Consequently, we conducted decomposition analyses for these variables.

Table 8 lists the result for the decomposition analysis of the gender gaps in financial behaviour. The observed gender gaps are explained by the differences in the responses for the explanatory variables (157.4%), and their interactions (-34.6%) while the overall endowment effects are not significant. Therefore, the unexplained parts are the major reasons for the gender difference. Among endowments effects, financial knowledge and employment status largely account for the gender difference.

**Table 8:** Decomposition of gender differences in financial behaviour.

Financial behaviour	Men: 15.343 (0.026) Women: 15.580 (0.025)	Difference: -0.238** (0.036)		
		Endowments	Coefficients	Interaction
Overall		0.054(0.029)	-0.374**(0.046)	0.082*(0.041)
Attribution		-22.8%	157.4%	-34.6%
Age		0.014(0.008)	-0.978(0.846)	0.011(0.010)
		25.4%	261.4%	12.8%
Age^2^		-0.016(0.009)	0.638(0.474)	-0.013(0.010)
		-29.6%	-170.4%	-15.2%
Occupation: employee/public official				
Self-employed		0.009(0.009)	-0.005(0.006)	-0.009(0.011)
		16.8%	1.4%	-11.0%
Part-time		-0.006(0.010)	-0.042(0.027)	0.026(0.017)
		-11.2%	11.1%	31.8%
Not working		-0.068**(0.019)	-0.137*(0.057)	0.072*(0.030)
		-124.6%	36.7%	87.2%
Others		0.000(0.001)	-0.000(0.005)	-0.000(0.000)
		0.8%	0.0%	0.0%
Education: high school or lower				
Junior college		-0.004(0.011)	0.021(0.032)	-0.012(0.018)
		-6.7%	-5.6%	-14.5%
University or higher		0.037*(0.018)	-0.018(0.025)	-0.017(0.024)
		67.4%	4.8%	-21.1%
Financial education		0.010*(0.004)	-0.002(0.008)	-0.001(0.005)
		17.7%	0.6%	-1.8%
Financial literacy		0.083**(0.008)	0.232**(0.075)	0.021**(0.007)
		152.7%	-62.1%	26.0%
Household income/100		-0.014**(0.004)	0.033(0.065)	0.003(0.006)
		-26.4%	-8.8%	3.6%
Financial assets/100		0.010(0.006)	0.068(0.045)	0.002(0.002)
		17.7%	-18.2%	2.2%
Constant			-0.183(0.390)	
			48.9%	
N		23,788 (Men: 11,658, Women: 12,130)		
Number of imputations		20		

a) coefficients, and robust standard errors in parentheses.

b) ** p<0.01, * p<0.05.

Table 9 lists the result for the decomposition analysis of the gender gaps in financial literacy. In line with financial attitude, the unexplained parts (i.e. coefficients effects and interaction effects) largely account for the gender difference.

**Table 9:** Decomposition of gender differences in financial attitude.

Financial attitude	Men: 9.410(0.025) Women: 9.947(0.025)	Difference: -0.537**(0.035)		
		Endowments	Coefficients	Interaction
Overall		0.084**(0.028)	-0.682**(0.045)	0.060(0.040)
Attribution		-15.7%	127.0%	-11.3%
Age		-0.019*(0.009)	-1.120(0.820)	0.012(0.010)
		-22.3%	164.3%	19.9%
Age^2^		0.028*(0.012)	1.021*(0.459)	-0.020(0.012)
		32.9%	-149.8%	-33.2%
Occupation: employee/public official				
Self-employed		-0.022*(0.009)	0.000(0.006)	0.001(0.011)
		-25.9%	-0.1%	1.4%
Part-time		0.032**(0.010)	-0.038(0.026)	0.024(0.016)
		38.1%	5.6%	39.7%
Not working		-0.033(0.018)	-0.121*(0.055)	0.063*(0.029)
		-38.9%	17.7%	104.4%
Others		-0.000(0.000)	0.005(0.005)	0.000(0.001)
		0.0%	-0.7%	0.8%
Education: high school or lower				
Junior college		-0.021*(0.010)	0.031(0.031)	-0.018(0.017)
		-24.5%	-4.6%	-29.4%
University or higher		0.032(0.017)	0.019(0.024)	0.018(0.023)
		37.9%	-2.8%	30.1%
Financial education		-0.007(0.004)	0.004(0.008)	0.003(0.005)
		-8.6%	-0.7%	4.7%
Financial literacy		0.094**(0.008)	-0.257**(0.072)	-0.024**(0.007)
		111.4%	37.7%	-39.1%
Household income/100		-0.011**(0.004)	-0.018(0.062)	-0.002(0.006)
		-13.2%	2.6%	-2.6%
Financial assets/100		0.011(0.007)	0.072(0.041)	0.002(0.002)
		13.1%	-10.6%	3.2%
Constant			-0.281(0.378)	
			41.3%	
N		23,788 (Men: 11,658, Women: 12,130)		
Number of imputations		20		

a) coefficients, and robust standard errors in parentheses.

b) ** p<0.01, * p<0.05.

### Robustness check

To check the robustness of our findings, we conduct additional analyses using a different measurement of financial literacy. Here, we define financial literacy by the ’Big 3’ questions [21] (i.e. No. 8, 10, and 14 in Table 1), which address numeracy, understanding of inflation, and knowledge about stocks and risk diversification. Although we find similar results to those from our more detailed measurements, the contribution of the differences in the distribution of the explanatory variables to gender gap in financial literacy is smaller, which may suggest that a small variation in the measurement based on only three questions may generate a measurement error (S1 and S2 Tables).

## Discussion

The purpose of this study was to investigate gender differences in financial literacy, as well as the association of financial literacy with individual characteristics (e.g. age and education). Our findings are twofold:

First, we find an inverse U-shaped relationship between financial literacy and age for both men and women. However, for self-rated financial knowledge, particularly for men, the opposite trend was observed: a U-shaped relationship with age was found. This trend is consistent with findings in many other countries [3]. The study indicates that younger and older people are likely to have lower financial literacy than those middle-aged. A recent study indicates that the decline in crystallised intelligence and fluid intelligence contributes to a corresponding decline in financial literacy [26]. Furthermore, among older people, while financial literacy declines, self-rated financial literacy remains high. As known as an ‘overconfidence bias,’ the miscalibration of self-assessment is explained by heuristics biases, the probabilistic mental model, and individual differences [27]. Among older people, self-confidence in financial management does not decline even though financial literacy drops in alignment with cognitive decline, potentially reflecting beliefs about accumulated experiences or reluctance to accept a decrease in cognitive functioning due to natural aging [28,29]. Although gender differences in the accuracy of self-assessment are inconclusive, the fact or stereotype that men are more financially literate than women may contribute to a larger magnitude of overconfidence among men [30,31].

Second, gender gaps in financial literacy are driven by differences in the distribution of the independent variables that impact financial literacy (e.g. education and the amount of financial assets). Previous research also suggests that women tend to be less financially literate than men both in the younger and older age groups [3]. This could be perpetuated by an underinvestment in financial literacy for women due to the stereotype that the financial capabilities of women are low [32] or that men and women acquire financial literacy differently [33]. However, no explicit reason had been confirmed. As women live longer than men, on average [34], they have to have a financial plan for managing their longer lives. Thus, strategies are needed that enhance financial literacy, particularly among women, and support financial decision making for a lifelong plan.

Our findings suggest that attaining higher education largely accounts for gender differences in financial literacy. It has been pointed out that those with higher educational are more financially literate, as financial literacy is affected by skills that those with higher education tend to possess potentially due to a positive association between financial literacy and cognitive skill [3]. Other research suggests that higher education is correlated positively with the ability to process information [35]. Therefore, gender differences could be mitigated through enhanced educational opportunities, including financial education for women.

However, financial behaviours and attitudes among women were more positive than men despite the better financial knowledge among men, in line with the previous findings [15]. This could be because the linkage of knowledge with behaviour and attitude is not always evident [36,37]. In addition, we find that the unexplained parts account for the gender gaps, as traditionally, women tend to be more risk averse and have the higher propensity to save [38–41]. Therefore, while enhancing financial knowledge is important, transforming it into actual behaviour and attitude is also necessary.

Our findings suggest two important policy implications. First, there is a need to support older adults regarding their financial decision making and for financial education for those with low financial literacy. If an individual remains confident, even when financial literacy declines, he/she may choose an inappropriate option or be subject to financial fraud without sufficient understanding of the option and support from advisors. Therefore, it is crucial that to provide appropriate assistance, financial planners in charge of support for financial decision making among individuals understand that preferences, abilities, and natures can change as people age. Simultaneously, individuals should be made aware that their financial literacy may decline as they age. Second, financial education and resources that individuals can access easily are required to ensure that necessary and timely information is available according to each life stage. These resources should be well organised so that individuals can accumulate knowledge that can enhance their actual financial behaviours and attitudes.

While our study investigates the determinants of financial literacy in Japan, focusing in particular on age and gender differences, it has several limitations. First, selection bias may exist, as the sample of the Financial Literacy Survey 2016 was collected through an internet survey. Thus, the surveyed sample may include those with higher IT literacy, especially among seniors. Furthermore, the respondents may be more interested than the general population in topics related to financial assets and decision making. Thus, the actual distribution of financial literacy among all Japanese people may have a lower mean and decrease more as a consequence of ageing. In fact, any sample of seniors randomly selected to measure financial literacy may be biased due to the decline in cognitive functioning. Unbiased methods to measure financial literacy, financial behaviour, and financial assets among a general sample, particularly among seniors, should be developed in future studies.

Second, as the Financial Literacy Survey 2016 is cross-sectional, it fails to follow any changes in financial literacy in the participant associated with ageing, thus, results may be subject to cohort differences rather than effects of age. Nevertheless, it would be unrealistic to study individuals from early to later life due to cost and sample attrition. Instead, it would be helpful to utilise panel data, even considering a relatively short follow-up period, to verify when and how individuals enhance their financial literacy and financial behavioural choices for better strategies related to financial asset management.

Additionally, endogeneity can be problematic in research on financial literacy and financial behavioural choices: as high financial literacy enables better financial behavioural choices, individuals might acquire literacy related to financial decision making when they have to make financial choices [3]. Moreover, unobserved factors, not considered in this study due to data restriction, may account for financial literacy and generate gender differences in financial literacy. Therefore, a further investigation should focus on: (1) causal impacts of financial literacy on financial behavioural choices and outcomes of actual asset management; (2) when and how individuals acquire financial literacy, and whether it improves financial behavioural choices; 3) if any other factors account for gender differences in financial literacy, behaviours, and attitudes. One previous work found that financial literacy, even among an educated group, was low [42]. However, concrete evidence for reasons why women are less financially literate than men seems difficult to find [3]; thus, accumulating such evidence is needed to enhance women’s financial literacy.

In conclusion, we found an inverse U-shaped relationship between age and financial literacy for both men and women, while the opposite trend appeared for age and self-rated financial knowledge, particularly among men. Furthermore, men were more financially literate than women and this difference could be explained by endowment effects, specifically higher education, coefficient effects, and interaction effects. Due to time and budget constraints, most studies on this topic utilise observational approaches. Hereafter, intervention studies and evidence-based policymaking will be required to support individuals in financial asset management to enable ‘the 100-Year Life’.

## Supporting information

S1 Table Estimation results for financial literacy measured by Big3 questions. (DOCX)

S2 Table Decomposition of gender differences in financial literacy measured by Big3 questions. (DOCX)

## References

[1] Financial Service Agency in Japan. About the results of verification of effects of NISA. https://www.fsa.go.jp/policy/nisa/20161021-1.html (**Accessed: 30 April, 2020**) (**in Japanese**)(2016).

[2] OECD. G20/OECD INFE report on adult financial literacy in G20 countries. (2017).

[3] Lusardi A. & Mitchell O. S. The Economic Importance of Financial Literacy: Theory and Evidence. *J*. *Econ*. *Lit*. 52, 5–44 (2014). doi: 10.1257/jel.52.1.5 28579637 PMC5450829

[4] Alessie R. O. B., Van Rooij M. & Lusardi A. Financial literacy and retirement preparation in the Netherlands. *Journal of Pension Economics and Finance* 10, 527–545 (2011).

[5] van Rooij M. C. J., Lusardi A. & Alessie R. J. M. Financial Literacy, Retirement Planning and Household Wealth*. *The Economic Journal* 122, 449–478 (2012).

[6] Behrman J. R., Mitchell O. S., Soo C. K. & Bravo D. How Financial Literacy Affects Household Wealth Accumulation. *American Economic Review* 102, 300–304 (2012). doi: 10.1257/aer.102.3.300 23355747 PMC3554245

[7] Bannier C. E. & Schwarz M. Gender- and education-related effects of financial literacy and confidence on financial wealth. *Journal of Economic Psychology* 67, 66–86 (2018).

[8] Monticone C. How Much Does Wealth Matter in the Acquisition of Financial Literacy? *Journal of Consumer Affairs* 44, 403–422 (2010).

[9] Klapper L. & Panos G. A. Financial literacy and retirement planning: the Russian case. *Journal of Pension Economics and Finance* 10, 599–618 (2011).

[10] Lusardi A. & Mitchell O. S. Financial literacy and retirement planning in the United States. *Journal of Pension Economics and Finance* 10, 509–525 (2011). 10.1017/S1474747211000448 PMC5445931 28553190

[11] Hsu J. W. Aging and strategic learning: The impact of spousal incentives on financial literacy. *Journal of Human Resources* 51, 1036–1067 (2016). doi: 10.3368/jhr.51.4.1014-6712r 28148971 PMC5279946

[12] Shimizutani, S. & Yamada, H. Financial Literacy of Middle and Older Generations: Comparison of Japan and the United States. *Keio-IES Discussion Paper Series* **DP 2018-016**(2018).

[13] Sekita S. Financial literacy and retirement planning in Japan. *Journal of Pension Economics and Finance* 10, 637–656 (2011).

[14] Yamori N. & Ueyama H. How Does the Financial Literacy Affect Borrowers’ Actions to Compare Mortgages Among Financial Institutions? *Studies in Financial Planning* 15, 4–12. (in Japanese) (2015).

[15] Kadoya Y. & Khan M. S. R. Financial Literacy in Japan: New Evidence Using Financial Knowledge, Behavior, and Attitude. *Sustainability* 12(2020).

[16] Yoshino, N., Morgan, P. & Trinh, L. Financial Literacy in Japan: Determinants and Impacts. *ADBI Working Paper* 796(2017).

[17] Saito Y. Gender Equality in Education: Japanese Experience. *NIER Research Bulletin* 143, 137–149 (2014).

[18] Gender Equality Bureau Cabinet Office. White paper on gender equality 2020. (**in Japanese**)(2020).

[19] Huston S. J. Measuring Financial Literacy. *Journal of Consumer Affairs* 44, 296–316 (2010).

[20] Potrich A. C. G., Vieira K. M. & Kirch G. Determinants of Financial Literacy: Analysis of the Influence of Socioeconomic and Demographic Variables. *Revista Contabilidade & Finanças* 26, 362–377 (2015).

[21] Lusardi A. & Mitchell O. S. Planning and Financial Literacy: How Do Women Fare? *American Economic Review* 98, 413–417 (2008).

[22] Atkinsoni, A. & Messyi, F. A. Measuring Financial Literacy: Results of the OECD / International Network on Financial Education (INFE) Pilot Study. *OECD Working Papers on Finance, Insurance and Private Pensions* (2012).

[23] Atkinsoni, A. & Messyi, F. Measuring Financial Literacy: Results of the OECD / International Network on Financial Education (INFE) Pilot Study. in *OECD Working Papers on Finance, Insurance and Private Pensions*, Vol. 15 (OECD Publishing, Paris, 2011).

[24] Blinder A. S. Wage Discrimination: Reduced Form and Structural Estimates. *The Journal of Human Resources* 8(1973).

[25] Oaxaca R. Male-Female Wage Differentials in Urban Labor Markets. *International Economic Review* 14(1973).

[26] Finke M. S., Howe J. S. & Huston S. J. Old Age and the Decline in Financial Literacy. *Management Science* 63, 213–230 (2017).

[27] Pallier G. Gender differences in the self-assessment of accuracy on cognitive tasks. *Sex Roles* 48, 265–276 (2003).

[28] Pak T.-Y. & Chatterjee S. Aging, overconfidence, and portfolio choice. *Journal of Behavioral and Experimental Finance* 12, 112–122 (2016).

[29] Gamble K., Boyle P., Yu L. & Bennett D. Aging and Financial Decision Making. *Manage Sci* 61, 2603–2610 (2015). doi: 10.1287/mnsc.2014.2010 26622068 PMC4662381

[30] Marsh H. W. & Yeung A. S. Longitudinal Structural Equation Models of Academic Self-Concept and Achievement: Gender Differences in the Development of Math and English Constructs. *American Educational Research Journal* 35, 705–738 (2016).

[31] Marsh H. W. A multidimensional, hierarchical model of self-concept: Theoretical and empirical justification. *Educ*. *Psychol*. *Rev*. 2, 77–172 (1990).

[32] Driva A., Lührmann M. & Winter J. Gender differences and stereotypes in financial literacy: Off to an early start. *Econ*. *Letters* 146, 143–146 (2016).

[33] Loibl C. & Hira T. K. A workplace and gender-related perspective on financial planning information sources and knowledge outcomes. *Financial Services Review* 15, 21 (2006).

[34] Austad S. N. & Fischer K. E. Sex Differences in Lifespan. *Cell Metab*. 23, 1022–1033 (2016). doi: 10.1016/j.cmet.2016.05.019 27304504 PMC4932837

[35] Pressley M., Borkwski J. G. & Schneider W. Good information processing: What it is and how education can promote it. *International Journal of Educational Research* 13, 857–867 (1989).

[36] Fabrigar L. R., Petty R. E., Smith S. M. & Crites S. L. Jr. Understanding knowledge effects on attitude-behavior consistency: the role of relevance, complexity, and amount of knowledge. *J*. *Pers*. *Soc*. *Psychol*. 90, 556–577 (2006). doi: 10.1037/0022-3514.90.4.556 16649855

[37] Walkey A. J., Law A. & Bosch N. A. Lottery-Based Incentive in Ohio and COVID-19 Vaccination Rates. *JAMA* 326(2021). doi: 10.1001/jama.2021.11048 34213530 PMC8385592

[38] Lee J. & Pocock M. L. Intrahousehold allocation of financial resources: evidence from South Korean individual bank accounts. *Review of Economics of the Household* 5, 41–58 (2007).

[39] Sapienza P., Zingales L. & Maestripieri D. Gender differences in financial risk aversion and career choices are affected by testosterone. *Proc Natl Acad Sci U S A* 106, 15268–15273 (2009). doi: 10.1073/pnas.0907352106 19706398 PMC2741240

[40] Seguino S. & Floro M. S. Does Gender have any Effect on Aggregate Saving? An empirical analysis. *Int*. *Rev*. *Appl*. *Econ*. 17, 147–166 (2003).

[41] Jianakoplos N. A. & Bernasek A. Are Women More Risk Averse? *Economic Inquiry* 36, 620–630 (1998).

[42] Mahdavi M. & Horton N. J. Financial Knowledge among Educated Women: Room for Improvement. *The Journal of Consumer Affairs* 48, 403–417 (2014).

