# Supplementary material for: Age, gender, and financial literacy in Japan
Source: PLoS One. 2021 Nov 17;16(11):e0259393. doi: 10.1371/journal.pone.0259393 (PMC8598074; doi:10.1371/journal.pone.0259393)
Supplement: S1 Table — (DOCX) [file pone.0259393.s001.docx]

S1 Table. Estimation results for financial literacy measured by Big3 questions

|  | (1) All | (2) Men | (3) Women |
| --- | --- | --- | --- |
| Age | -0.300** | 0.030** | 0.048** |
|  | (0.015) | (0.005) | (0.004) |
| Age^2^ | 0.037** | -0.000** | -0.000** |
|  | (0.003) | (0.000) | (0.000) |
| Male | -0.000** |  |  |
|  | (0.000) |  |  |
| Occupation: employee/public official | Reference | | |
| Self-employed | -0.063* | -0.023 | -0.136** |
|  | (0.027) | (0.033) | (0.050) |
| Part-time | -0.125** | -0.102** | -0.163** |
|  | (0.022) | (0.039) | (0.028) |
| Not working | -0.074** | 0.010 | -0.136** |
|  | (0.019) | (0.032) | (0.026) |
| Others | -0.055 | -0.055 | -0.066 |
|  | (0.047) | (0.065) | (0.068) |
| Education: high school or lower | Reference | | |
| Junior college | 0.076** | 0.048 | 0.096** |
|  | (0.018) | (0.032) | (0.022) |
| University or higher | 0.343** | 0.308** | 0.398** |
|  | (0.016) | (0.023) | (0.024) |
| Financial education | 0.141** | 0.051 | 0.284** |
|  | (0.029) | (0.037) | (0.045) |
| Household income/100 | 0.008** | 0.013** | 0.006 |
|  | (0.003) | (0.004) | (0.004) |
| Financial assets/100 | 0.031** | 0.028** | 0.033** |
|  | (0.001) | (0.002) | (0.002) |
| Constant | 0.240** | 0.484** | -0.355** |
|  | (0.071) | (0.110) | (0.093) |
| N | 23,788 | 11,658 | 12,130 |
| Number of imputations | 20 | | |

a) coefficients, and robust standard errors in parentheses.

b) ** p<0.01, * p<0.05.
