# Supplementary material for: Age, gender, and financial literacy in Japan
Source: PLoS One. 2021 Nov 17;16(11):e0259393. doi: 10.1371/journal.pone.0259393 (PMC8598074; doi:10.1371/journal.pone.0259393)
Supplement: S2 Table — (DOCX) [file pone.0259393.s002.docx]

S2 Table 2. Decomposition of gender differences in financial literacy measured by Big3 questions

| Financial Literacy | Men: 1.907 (0.010)  Women: 1.489 (0.010) | Difference | | |
| --- | --- | --- | --- | --- |
|  |  | 0.419** (0.014) | | |
|  | | Endowments | Coefficients | Interaction |
| Overall | | 0.150**(0.012) | 0.328**(0.017) | -0.059**(0.015) |
| Attribution | | 35.8% | 78.3% | -14.1% |
| Age | | -0.026**(0.010) | -0.928**(0.316) | 0.010*(0.005) |
|  | | -17.3% | -282.9% | -16.9% |
| Age^2^ | | 0.017*(0.007) | 0.373*(0.177) | -0.007(0.004) |
|  | | 11.3% | 113.7% | 11.9% |
| Occupation: employee/public official | |  |  |  |
| Self-employed | | -0.009**(0.003) | 0.004(0.002) | 0.008(0.004) |
|  | | -6.0% | 1.2% | -13.6% |
| Part-time | | 0.022**(0.004) | 0.013(0.010) | -0.008(0.006) |
|  | | 14.7% | 4.0% | 13.6% |
| Not working | | 0.037**(0.007) | 0.075**(0.021) | -0.039**(0.011) |
|  | | 24.7% | 22.9% | 66.1% |
| Others | | -0.000(0.000) | 0.000(0.002) | 0.000(0.000) |
|  | | -0.0% | 0.0% | 0.0% |
| Education: high school or lower | |  |  |  |
| Junior college | | -0.017**(0.004) | -0.015(0.012) | 0.009(0.007) |
|  | | -11.3% | -4.6% | -15.3% |
| University or higher | | 0.109**(0.007) | -0.026**(0.009) | -0.025**(0.009) |
|  | | 72.7% | -7.9% | 42.4% |
| Financial education | | 0.010**(0.002) | -0.013**(0.003) | -0.008**(0.002) |
|  | | 6.7% | -4.0% | 13.6% |
| Household income/100 | | 0.003(0.002) | 0.035(0.024) | 0.003(0.002) |
|  | | 6.7% | -4.0% | 13.6% |
| Financial assets/100 | | 0.005(0.004) | -0.031(0.016) | -0.001(0.001) |
|  | | 3.3% | -9.5% | 1.7% |
| Constant | |  | 0.839**(0.146) |  |
|  | |  | 255.8% |  |
| N | | 23,788 (Men: 11,658, Women: 12,130) | | |
| Number of imputations | | 20 | | |

a) coefficients, and robust standard errors in parentheses.

b) ** p<0.01, * p<0.05.
